# Effectiveness of treatments to prevent femoral periprosthetic bone loss following total hip arthroplasty: a network meta-analysis

**DOI:** 10.3389/fphar.2025.1566890

**Published:** 2025-11-27

**Authors:** Zhi-Hu Zhao, Tao Ling, Songqing Ye, Wei Luo, Jian-Xiong Ma, Xinlong Ma

**Affiliations:** 1 Department of Orthopaedics, Tianjin Hospital, Tianjin, China; 2 Department of Pharmacy, Suqian First Hospital, Suqian, China; 3 Tianjin Institute of Orthopedics in Traditional Chinese and Western Medicine, Tianjin, China

**Keywords:** total hip arthroplasty, meta-analysis, bone mineral density, network, anti-osteoporosis

## Abstract

**Background:**

The objective of this study was to conduct a systematic review and network meta-analysis (NMA) to compare the efficacy of various anti-osteoporosis drugs in preventing femoral periprosthetic bone loss following total hip arthroplasty (THA).

**Methodology:**

Randomized controlled trials (RCTs) assessing the clinical efficacy of various anti-osteoporosis drugs and control treatments in preventing periprosthetic bone loss following THA were identified. Outcomes evaluated included bone mineral density (BMD) at 6 months, 12 months, 24 months, and 5–10 years. The network meta-analysis was conducted using Stata 13.0 and R-3.5.1 software with the “gemtc” package.

**Results:**

A total of 33 RCTs with 1,169 patients were included. At 6 months, alendronate, alendronate + alfacalcidol, denosumab, ibandronate, raloxifene, teriparatide + alendronate, and zoledronic acid were beneficial in increasing BMD, with denosumab ranking highest (based on surface under the cumulative ranking curve (SUCRA) values). At 12 months, alendronate, alendronate + alfacalcidol, denosumab, ibandronate, risedronate, and zoledronic acid showed benefits, with alendronate + alfacalcidol ranking highest (SUCRA = 0.97). For 24-month BMD, teriparatide + alendronate ranked highest (SUCRA = 0.82). Analysis of BMD at 5–10 years, involving four studies on alendronate, pamidronate, and placebo, indicated that alendronate achieved the highest SUCRA value (0.87).

**Conclusion:**

Both denosumab and bisphosphonates are effective in preventing femoral periprosthetic bone loss following THA. Denosumab was the most efficient agent for increasing BMD at 6 months post-THA, while alendronate combined with alfacalcidol or feriparatide was most efficient at 12 months and 24 months. More high-quality direct comparisons and long-term follow-up studies are needed to determine the optimal drug and dosage for THA patients.

## Introduction

Total hip arthroplasty (THA) is a widely performed surgical procedure designed to relieve pain and improve quality of life for individuals with conditions like osteoarthritis and osteonecrosis of the femoral head (ONFH) (Gao et al., 2017; Anderl et al., 2022; Knutsen et al., 2017). In the United States alone, more than 230,000 primary THA surgeries are performed annually, with the global figure exceeding 500,000. This number is expected to rise by approximately 10% each year (Su et al., 2021; Sariali et al., 2020). Despite its success, the long-term durability of THA is often compromised by progressive bone loss around the implant. Studies report that bone mineral density (BMD) in the proximal femur can decline by up to 40% within the first year after surgery compared to immediately post-operation (Yan et al., 2017; Gerhardt et al., 2019). This bone loss is believed to contribute to aseptic loosening and late-stage implant-related fractures, posing significant challenges to implant longevity (Zhang et al., 2018; Tapaninen et al., 2012). Consequently, various strategies, such as the utilization of bisphosphonates, denosumab, and anabolic osteoporosis drugs, have been employed to preserve bone mass and enhance the stability of the implant, ultimately leading to improved outcomes in THA (Tran et al., 2016).

Bisphosphonates, which are antiresorptive agents, can bind to hydroxyapatite and inhibit farnesyl-pyrophosphate synthase, an enzyme involved in the mevalonate pathway. These agents are frequently utilized in the management of osteoporosis and various metabolic bone disorders (Wang et al., 2018). Recent research has indicated that bisphosphonates possess the potential to decrease the likelihood of fractures and prolong the lifespan of implants (Shi et al., 2018a). Numerous studies, including randomized controlled trials (RCTs), have been conducted to explore the effects of bisphosphonates on the preservation of periprosthetic bone mineral density following THA (Shi et al., 2018b; Lin et al., 2012; Bhandari et al., 2005).

Multiple meta-analyses have been conducted to compare the efficacy of bisphosphonates versus control in mitigating bone loss subsequent to THA (Shi et al., 2018b; Zhao et al., 2015; Di Martino et al., 2024; Hatano et al., 2025). However, the indiscriminate amalgamation of outcomes with differing levels of evidence may undermine the obtained effect size. Additionally, the aggregation of results from diverse follow-up periods without proper categorization diminishes the reliability of the findings. Apart from qualitative analyses, the literature only provides two meta-analyses that compare bisphosphonates for bone loss following THA and total knee arthroplasty (Shi et al., 2018b; Bhandari et al., 2005). Regrettably, the aforementioned studies failed to distinguish between THA and total knee arthroplasty, resulting in ambiguity during the interpretation process.

Denosumab, a fully humanized monoclonal antibody, acts as an osteoprotegerin (OPG) mimicker by targeting the receptor activator of nuclear factor kappa B ligand (RANKL), which influences the activity, survival, and recruitment of osteoclasts (Deeks, 2018). In a network meta-analysis encompassing ten drugs, denosumab exhibited the most effective efficacy in preventing and tolerating BMD loss in the hip and femur (Migliorini et al., 2021).

Anabolic osteoporosis medications, such as teriparatide, are typically prescribed exclusively for patients who have confirmed and severe osteoporosis (Dong et al., 2024; Roy and Mazumdar, 2023). Teriparatide, a synthetic form of human parathyroid hormone (PTH) (1–34), is the sole authorized anabolic therapy for osteoporosis in multiple countries (Mineta et al., 2025). Research has demonstrated its efficacy in reducing the likelihood of both vertebral and nonvertebral fractures in individuals with osteoporosis induced by glucocorticoids (Liu et al., 2020).

However, a significant proportion of these articles have been subjected to placebo comparisons, while direct comparisons between different pharmacological interventions, including the utilization of innovative drugs, have been lacking. Moreover, the scarcity of evidence and the absence of direct statistical analysis pose challenges for physicians in determining the most effective treatment.

A network meta-analysis (NMA) is a methodological approach that facilitates the simultaneous comparison of multiple treatments within a single meta-analysis (Shim et al., 2019). In line with this approach, the present systematic review and NMA aim to compare different anti-osteoporosis drugs in terms of femoral periprosthetic bone loss following THA. Through the implementation of this network meta-analysis, it becomes possible to directly compare and forecast the most efficacious anti-osteoporosis drugs following THA.

## Methods

This systematic review was written according to the PRISMA (Preferred Reporting Items for Systematic Reviews and Meta-analyses) checklist and A Measurement Tool to Assess Systematic Reviews 2 (AMSTAR2) (Shea et al., 2007). This systematic review protocol has been registered on PROSPERO (www.crd.york.ac.uk/prospero/) with the number CRD42023453329 (Page et al., 2021).

### Search strategy

**FIGURE 1:**
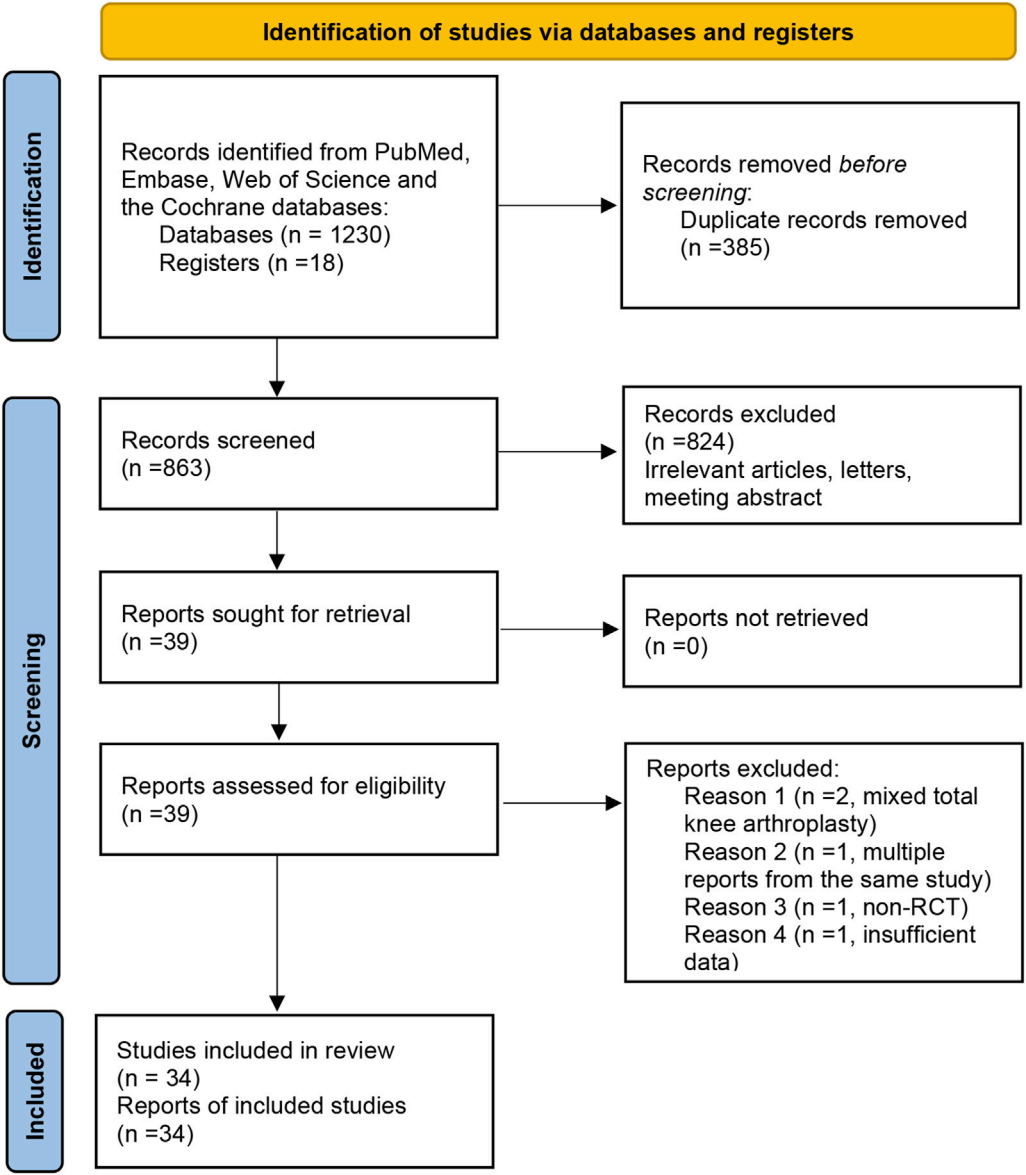
Flow diagram of the literature selection process.

The first two authors (Zhi-hu Zhao and Tao Ling) independently searched the PubMed, Embase, Web of Science, and the Cochrane databases from the date of their inception to November 2024. Reference lists of previously published systematic reviews were also reviewed for potentially relevant studies. The terms used for screening THA were “THA or THR OR total hip arthroplasty OR total hip replacement OR ‘Arthroplasty, Replacement, Hip’ [Mesh]”, based on a previous systematic review (Shi et al., 2018b). The terms used for screening bisphosphonates were “alendronate OR pamidronate OR etidronate OR zoledronate OR clodronate OR bisphosphonate OR statin”. The term “alfacalcidol OR risedronate OR teriparatide OR denosumab OR simvastatin” was added in the searching process to identify other relevant studies. To enhance the scope of the search for pertinent studies, the terms “BMD,” “bone density,” “osteoporosis,” and “periprosthetic fracture” were employed in querying relevant databases. Keywords and medical subject headings (MeSH) terms were used to identify relevant literature. All retrieved citations were imported into EndNote X7 software (EndNote Clarivate Analytics, USA), a reference management software, for deduplication and organization. The subsequent screening process was conducted using this software to manage the literature and generate the final reference list. A flow diagram of the literature selection process can be seen in Figure 1.

### Inclusion and exclusion criteria

The inclusion criteria: (1) all studies included in this NMA were RCTs, irrespective of the language or publication status; (2) Participants were adult patients (typically over 20 years of age) who underwent primary total hip arthroplasty (THA); (3) Studies using any of the following interventions were included: alendronate, etidronate, zoledronic acid, alfacalcidol, alendronate + alfacalcidol, risedronate teriparatide, denosumab pamidronate, clodronate, simvastatin ibandronate and placebo; (4) The BMD around the stem was assessed at least 6 months after THA.

The exclusion criteria: (1) non-randomized controlled study; (2) patients in the study were revision THA or had a metabolic bone disorder other than osteoporosis; (3) studies with insufficient data. The full texts of all included articles were obtained via institutional library access or the authors’ user profiles on the ResearchGate platform.

### Study selection

To complete the study selection, two authors (Songqing Ye and Wei Luo) utilized EndNote (Thomson Reuters, New York, NY, United States) to organize all studies. First, the “remove duplicates” command was used, and duplicates were removed. We then reviewed abstracts of the remaining records to remove studies based on the inclusion and exclusion criteria. Finally, the full texts of potentially eligible studies were retrieved and reviewed by two independent reviewers (Jian-xiong Ma and Wei Luo), and any discrepancies during the selection process were resolved by Xinlong Ma.

### Data extraction

The first two authors (Zhi-hu Zhao and Tao Ling) independently reviewed all titles and abstracts. In case of inadequate information, the full text was retrieved for further screening. The same authors independently extracted data from eligible studies, including the name of the first author, treatment, control, sample size of treatment and control, mean age of the patients, surgery, intervention duration, follow-up, results, and level of evidence. Primary outcomes were periprosthetic BMD at 6 months, 12 months, 24 months, and 5–10 years. Dual-energy X-ray absorptiometry (DXA) was used to measure periprosthetic BMD in the entire hip. To reduce bias from varying baseline data time points, BMD (g/cm^2^) was used instead of percentage change in BMD.

### Quality assessment

The first two reviewers (Zhi-hu Zhao and Tao Ling) independently used the Cochrane’s risk of bias tool to evaluate the methodological quality of the selected RCTs. A low, unclear, or high risk of bias value was assigned to the following items: random sequence generation, allocation concealment, blinding of participants and personnel, blinding of outcome assessment, incomplete outcome data, selective reporting, and other bias. At the end of quality assessment, a consensus on the final evaluation was reached; any disagreements were resolved by the judgment of another author. Kappa values were used to measure the degree of agreement between the two reviewers and were rated as follows: fair, 0.40 to 0.59; good, 0.60 to 0.74; and excellent, 0.75 or more (Higgins et al., 2011).

### Assessing the transitivity assumption in network meta-analysis

A fundamental concept in network meta-analysis is the notion that all patients within a network should, in principle, be equally eligible to receive any of the available treatments. This principle is often referred to as “jointly randomizable” (Ahn and Kang, 2021). Essentially, it implies that all patients within our network should, theoretically, have the option to receive any of the anti-osteoporotic agents described in the analysis. To assess the validity of the transitivity assumption, which underpins the network meta-analysis, we evaluated whether the participants in the identified studies were jointly randomizable. We also determined whether effect modifiers were distributed consistently across different treatment options in the network (Efthimiou et al., 2016). We tested the distribution of commonly espoused effect modifiers (i.e., publication year, age, and sample size) to ensure that they were balanced and that our estimates were not confounded.

### Statistically analysis

Time points of interest included 6 months, 12 months, 24 months, and 5–10 years postoperatively. In the absence of standard deviation (SD), the median value of SDs from other studies in the same comparison was borrowed. For data described as median and range, mean and SD were estimated according to a previous manner (Hozo et al., 2005). Weighted mean difference (WMD) and 95% credible intervals (CrI) were used as pooled effect size measures. Indirect network meta-analysis (NMA) was conducted on the “gemtc” and “rjags” packages in R (version 3.5.1, https://www.r-project.org/) (van Valkenhoef et al., 2012). A Bayesian random-effects analysis, which was based on the Markov chain Monte Carlo (MCMC) simulation from the posterior distribution, was adopted to calculate the estimates of relative effects and all model parameters. The probability of the best treatment was then ranked based on NMA results using the surface under the cumulative ranking curve (SUCRA). The larger the SUCRA, the higher the treatment in the hierarchy for an outcome. To evaluate the heterogeneity, the mtc.anohe command of the R package of “gemtc” was utilized by reporting the heterogeneity variance parameter I^2^. I^2^ > 50% was regarded as significant heterogeneity, and the random-effects model was utilized; otherwise, the fixed-effect model was utilized. To measure the consistency of results between direct and indirect meta-analyses, the node-splitting method or a comparison of effect sizes obtained from direct and indirect results was applied when applicable (Dias et al., 2010).

We performed a random-effects network meta-regression within a Bayesian hierarchical framework using the gemtc package in R. The gemtc package automatically determines uninformative prior distributions for all parameters in our model, which are commonly applied in NMA. We ran the Markov chain Monte Carlo simulation with four chains for each model, using 100,000 iterations, a burn-in of 5000 iterations, and extraction of every 10th value. We assessed convergence with the Gelman–Rubin–Brooks plot and potential scale reduction factor (a threshold of <1.05 indicates adequate convergence). We specified our assumptions (common or exchangeable covariate-comparison interaction) for each network meta-regression model once the data were available so as to make best use of the available data. We assumed coherent relative treatment effects estimated at the covariate value 0 and coherent regression coefficients for the treatment effect by covariate interaction. The network plot and funnel plots were generated using STATA 13.0 software (StataCorp LLC, College Station, TX, USA).

## Results

A total of 1,248 titles were obtained by primary search, including 1230 electronic records and 18 additional records from other sources. A total of 855 records were identified after duplicates were removed. Finally, 33 studies with 1,169 patients were included (Zhang et al., 2018; Arabmotlagh et al., 2009; Arabmotlagh et al., 2006; Arnala, 2012; Aro et al., 2018; Fokter et al., 2005; Gong et al., 2020; Hennigs et al., 2002; Huang et al., 2017; Iwamoto et al., 2011; Iwamoto et al., 2014; Kinov et al., 2006; Kobayashi et al., 2016; Morita et al., 2020; Muren et al., 2015; Nagoya et al., 2018; Nehme et al., 2003; Nishioka et al., 2007; Nyström et al., 2020; Scott et al., 2013; Shetty et al., 2006; Skoldenberg et al., 2011; Tapaninen et al., 2010; Trevisan et al., 2010; Venesmaa et al., 2001; Wilkinson et al., 2005; Yamaguchi et al., 2004; Yamaguchi et al., 2003; Yamasaki et al., 2007; Yang et al., 2019; Yukizawa et al., 2017; Zhou et al., 2019; Yamaguchi et al., 2005). Nine RCTs compared alendronate to placebo for BMD loss after THA. One RCT compared etidronate to placebo for BMD loss after THA. Three studies compared zoledronic acid to a placebo for BMD loss after THA. Five studies compared risedronate to placebo for BMD loss after THA. One study compared simvastatin to a placebo for BMD loss after THA. One study compared alendronate to teriparatide for BMD loss after THA. One study compared denosumab to placebo for BMD loss after THA. One study compared pamidronate to a placebo for BMD loss after THA. One study compared ibandronate and clodronate to a placebo for BMD loss after THA. Follow-up duration ranged from 6 months to 10 years (Table 1).

**TABLE 1:** General characteristics of the included studies. NS, not stated.

Author	Treatment	Control	Sample size (n)		Mean age (year)	Surgery	Intervention duration	Follow-up	Results	Level of evidence
			Treatment	Control						
Arabmotlagh 2009	Alendronate	Placebo	29	20	58.8	Uncemented THA	10 mg daily or 20 mg daily	6 years	Periprosthetic BMD, pain score, hip function	I
Arabmotlagh 2006	Alendronate	Placebo	27	24	64.2	Uncemented THA	13 patients for 4 months and 14 patients for 6 months	1 year	Periprosthetic BMD, serum bone alkaline phosphatase (BAP), osteocalcin, CTX-I	II
Fokter 2006	Etidronate	Placebo	18	20	68	Cemented THA	12 months	1 year	Periprosthetic BMD	I
Hennigs 2002	Alendronate	Placebo	42	24	52.9	Uncemented THA	12 months	1 year	Periprosthetic BMD	I
Huang 2017	Zoledronic acid	Placebo	15	15	60.1	Uncemented THA	2 years	2 years	Periprosthetic BMD, serum BAP, and serum NTX	I
Iwamoto 2011	Alendronate/alfacalcidol	Placebo	18/14	22	65	Uncemented THA	12 months	12 months	Periprosthetic BMD	I
Iwamoto 2014	Alendronate/alfacalcidol	Placebo	42	22	65	Uncemented THA	12 months	12 months	Periprosthetic BMD	II
Kinov 2006	Risedronate	Placebo	12	12	57.4	NS	6 months	6 months	Periprosthetic BMD, serum BAP, serum NTX	II
Kobayashi 2016	Alendronate	Teriparatide/Placebo	14	16	65	Uncemented THA	12 months	12 months	Periprosthetic BMD, lumbar BMD, serum P1NP, serum NTX	I
Muren 2015	Risedronate	Placebo	30	31	62	Uncemented THA	6 months	6 months	Periprosthetic BMD, femoral stem migration, hip function, adverse events	II
Nagoya 2018	Denosumab	Placebo	10	10	79.4	Uncemented THA	12 months	12 months	Periprosthetic BMD	II
Nehme 2003	Alendronate	Placebo	20	18	60	Cemented THA	24 months	24 months	Periprosthetic BMD	I
Nishioka 2007	Alendronate	Placebo	8	9	66.4	Uncemented THA	12 months	12 months	Periprosthetic BMD, lumbar spine BMD, serum calcium, serum phosphorus, deoxypyridinoline	II
Scott 2013	Zoledronic acid	Placebo	33	33	64.7	Uncemented THA	24 months	24 months	Periprosthetic BMD	I
Shetty 2006	Pamidronate	Placebo	23	24	58	Cemented THA	Once on the fifth postoperative day	5 years	Periprosthetic BMD	I
Skoldenberg 2011	Risedronate	Placebo	36	37	61	Uncemented THA	6 months	24 months	Periprosthetic BMD, femoral stem migration, hip function	I
Tapaninen 2010	Alendronate	Placebo	7	9	65	Uncemented THA	6 months	5 years	Periprosthetic BMD	I
Trevisan 2010	Clodronate	Placebo	50	54	63.7	Uncemented THA	12 months	12 months	Periprosthetic BMD, serum BAP, serum NTX	II
Venesmaa 2001	Alendronate	Placebo	8	5	62.5	Uncemented THA	6 months	6 months	Periprosthetic BMD	II
Yamaguchi 2004	Etidronate	Placebo	65	31	64	Uncemented THA	12 months	24 months	Periprosthetic BMD, serum BAP, urinary NTX	II
Yamasaki 2007	Risedronate	Placebo	19	21	53.4	Uncemented THA	6 months	6 months	Periprosthetic BMD	II
Yukizawa 2017	Alendronate	Alfacalcidol/placebo	20	40	63	Uncemented THA	2 years	10 years	Periprosthetic BMD, lumbar BMD	I
Zhang 2018	Simvastatin	Placebo	21	21	69.6	Uncemented THA	12 months	12 months	Periprosthetic BMD	II
Zhou 2019	Zoledronic acid	Placebo	20	20	73.8	NS	12 months	12 months	Periprosthetic BMD	I
Yang 2019	Ibandronate	Placebo	50	50	58.3	Uncemented THA	6 months	6 months	Periprosthetic BMD	I
Arnala 2012	Calcitonin	Placebo	30	30	NS	Cemented THA	12 months	12 months	Periprosthetic BMD	I
Gong 2020	Raloxifene	Placebo	120	120	62.5	Uncemented THA	prescribed	2 years	Periprosthetic BMD	I
Aro 2018	Zoledronate	Placebo	25	24	65.3	Uncemented THA	4 years	4 years	Periprosthetic BMD	I
Morita 2020	Teriparatide + Alendronate	Alendronate	14	12	65.8	Uncemented THA	24 months	1 year	Periprosthetic BMD	II
Nystrom 2020	Denosumab	Placebo	16	15	58	Uncemented THA	24 months	2 years	Periprosthetic BMD	I
Yamaguchi 2003	Etidronate	Placebo	22	30	70	Uncemented THA	12 months	1 year	Periprosthetic BMD	I
Fokter 2005	Etidronate	Placebo	26	20	70	Cemented THA	12 months	1 year	Periprosthetic BMD	I
Wilkinson 2005	Pamidronate	Placebo	22	22	57	Hybrid-type THA	24 months	2 years	Periprosthetic BMD	I
Yamaguchi 2005	Etidronate	Placebo	26	17	68	Uncemented THA	12 months	1 year	Periprosthetic BMD	I

**FIGURE 2:**
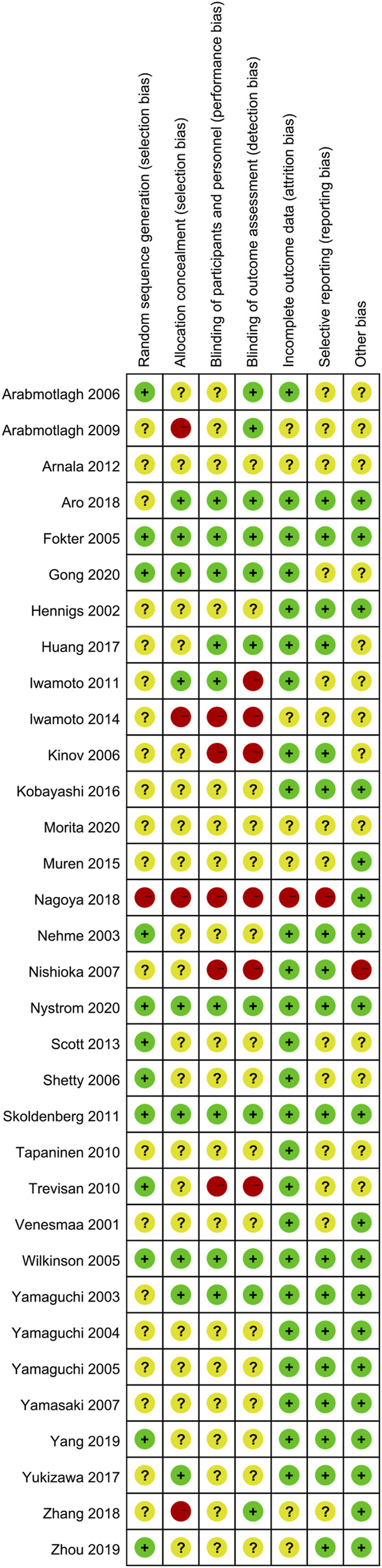
Risk-of-bias summary of the included 34 RCTs.

**FIGURE 3:**
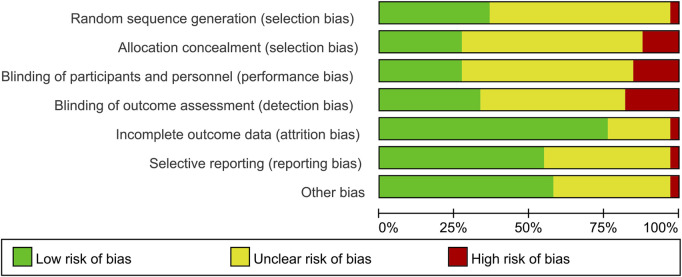
Risk-of-bias graph of the included studies.

Only 12 studies reported random sequence generation, which was classified as low risk of bias. Among the 34 studies included, one was evaluated as “high risk” in terms of selection bias related to random sequence generation. The remaining studies were categorized as having an unclear risk of bias. In terms of selection bias associated with allocation concealment, 26.5% of the studies were classified as low risk, 11.8% as high risk, and 60.5% as unclear risk of bias. Blinding of participants and personnel emerged as the most commonly identified factor associated with potential bias, with 10 studies deemed to have a low risk of bias due to participant blinding (29.4%). Additionally, a total of 12 studies were identified as having a low risk of bias for outcome assessment blinding (35.3%). A total of seven studies were classified as unclear risk of bias for incomplete outcome data. A total of eight studies were classified as low risk of bias for selective reporting, and 20 studies were classified as low risk of bias for other bias (Figures 2, 3). The overall kappa value regarding the evaluation of risk of bias of included RCTs was 0.827, indicating an excellent degree of agreement between the two reviewers.

### Assessment of transitivity

After grouping the studies by treatment comparison and inspecting the distribution of possible effect modifiers, there were no significant differences between the demographic characteristics for all treatments (Supplementary Figure S1). Therefore, they were judged to be sufficiently similar to be jointly synthesized in a network meta-analysis.

### Network structure diagrams

**FIGURE 4:**
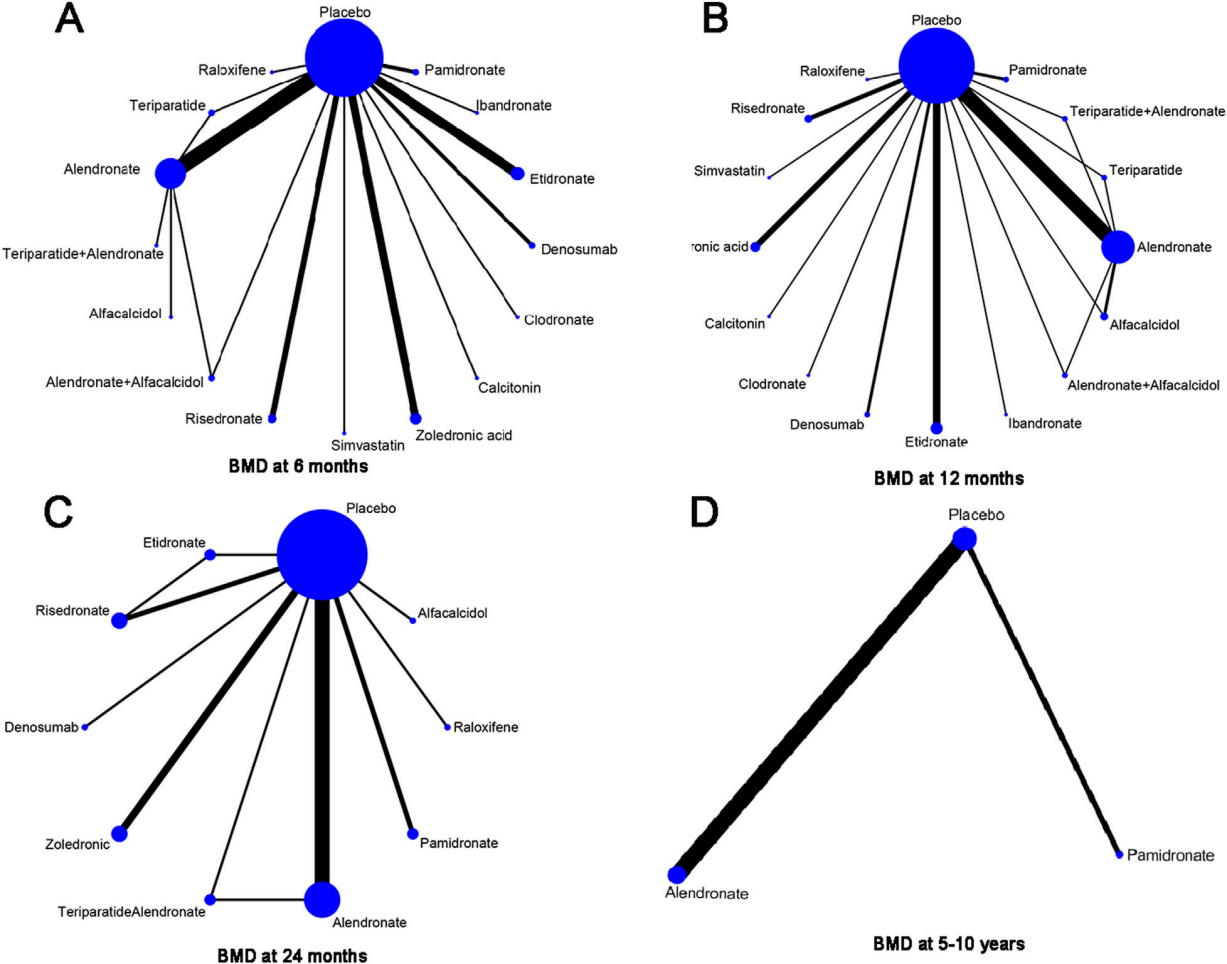
Network structure diagrams. **(A)** BMD at 6 months; **(B)** BMD at 12 months; **(C)** BMD at 24 months; and **(D)** BMD at 5–10 years. The numbers showed the number of direct comparisons. Line thicknesses are proportional to the number of direct comparisons. Circle diameters are proportional to the treatment numbers.

In this article, 15 different therapeutic drugs, namely, alendronate, alendronate + alfacalcidol, alfacalcidol, calcitonin, clodronate, denosumab, etidronate, ibandronate, pamidronate, raloxifene, risedronate, simvastatin, teriparatide, teriparatide + alendronate, zoledronic acid, and placebo, were finally enrolled. Four clinical outcomes comprising BMD at 6 months, 12 months, 24 months, and 5–10 years were ultimately evaluated. As displayed in Figure 4, the network structure diagrams detail the direct comparisons between different drugs in the four clinical outcomes, respectively. In addition, the numbers show the number of direct comparisons. Line thicknesses are proportional to the number of direct comparisons. Circle diameters are proportional to the number of patients treated who were included in this network meta-analysis.

### BMD at 6 months

**FIGURE 5:**
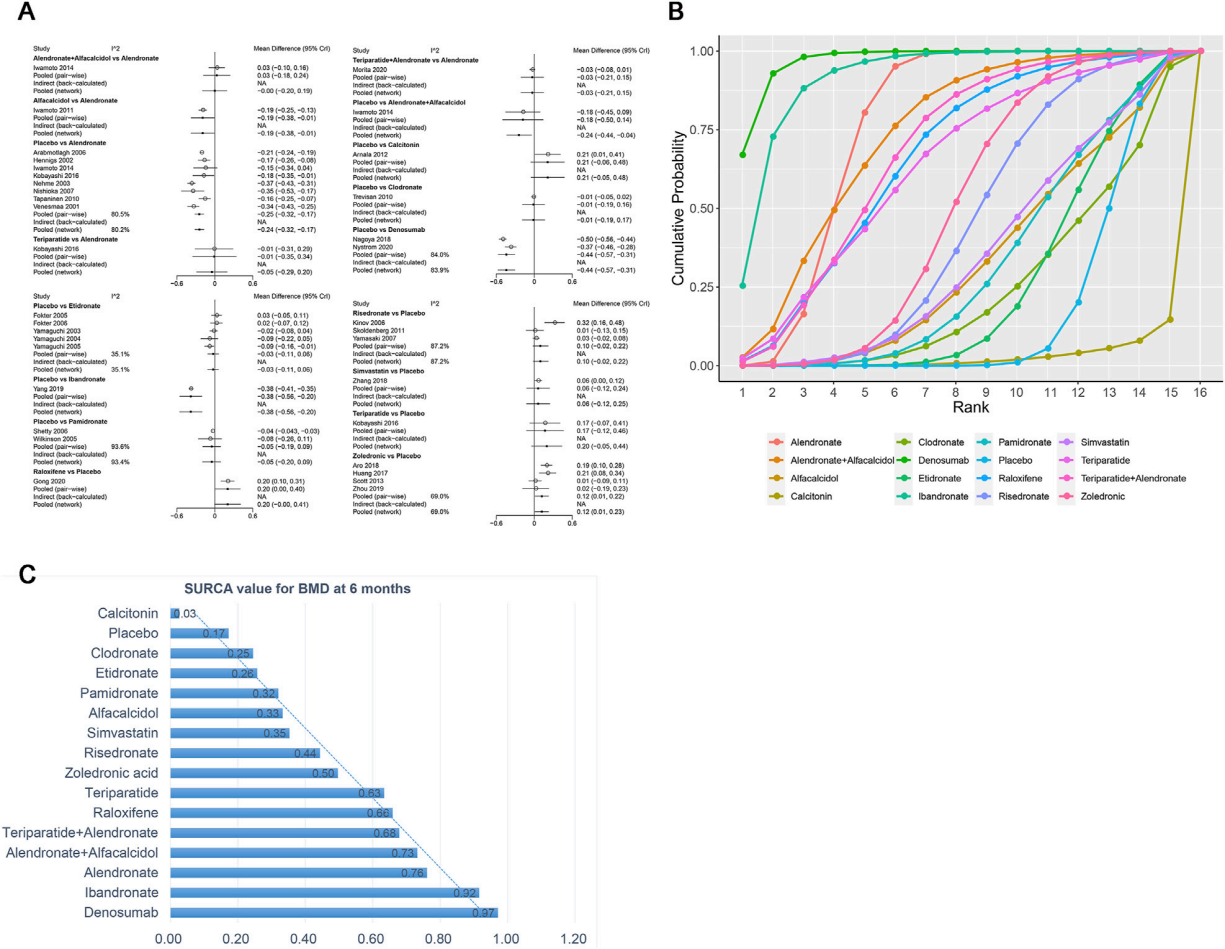
**(A)** Heterogeneity between different comparisons of drugs for BMD at 6 months. **(B)** Surface under the cumulative ranking curve (SUCRA) probabilities of different drugs for BMD at 6 months. **(C)** SUCRA values of the different drugs for BMD at 6 months.

A total of 31 RCTs, including 15 drugs (alendronate, alendronate + alfacalcidol, alfacalcidol, calcitonin, clodronate, denosumab, etidronate, ibandronate, pamidronate, raloxifene, risedronate, simvastatin, teriparatide, teriparatide + alendronate, zoledronic acid, and placebo) contributed to the clinical outcome of the BMD at 6 months. The network meta-analysis demonstrated that, compared with the control group, alendronate (WMD = 0.24, 95% CrI: 0.17, 0.32), alendronate + alfacalcidol (WMD = 0.24, 95% CrI: 0.04, 0.44), denosumab (WMD = 0.44, 95% CrI: 0.31, 0.57), ibandronate (WMD = 0.38, 95% CrI: 0.20, 0.56), raloxifene (WMD = −0.2, 95% CrI: 0.00, 0.40), teriparatide + alendronate (WMD = 0.21, 95% CrI: 0.02, 0.40), and zoledronic acid (WMD = 0.12, 95% CrI: 0.01, 0.23) exhibited a beneficial role in increasing the BMD at 6 months (Table 2). Figure 5A summarizes the heterogeneity between different comparisons of drugs. There was high heterogeneity between alendronate and placebo (I^2^ = 80.5%), pamidronate and placebo (I^2^ = 93.6%), denosumab and placebo (I^2^ = 83.9%), risedronate and placebo (I^2^ = 87.2%), and zoledronic acid and placebo (I^2^ = 69.0%). Thus, we applied the random-effect model for the cumulative values. As shown in Figures 5B,C, the SUCRA values of the five interventions demonstrated that denosumab ranked the highest SUCRA values of the BMD at 6 months (0.97).

**TABLE 2:** Efficacy of different comparisons of drugs by WMDs and corresponding 95% CrIs for BMD at 6 months. Bold fonts indicate p-value <0.05.

Placebo															
**−0.24 (−0.32, –0.17)**	Alendronate														
**−0.24 (−0.44, −0.04)**	0 (−0.19, 0.2)	Alendronate + alfacalcidol													
−0.05 (−0.25, 0.15)	**0.19 (0.01, 0.38)**	0.19 (−0.08, 0.45)	Alfacalcidol												
0.21 (−0.06, 0.48)	**0.46 (0.18, 0.73)**	**0.45 (0.12, 0.78)**	0.26 (−0.07, 0.59)	Calcitonin											
−0.01 (−0.19, 0.17)	**0.23 (0.04, 0.42)**	0.23 (−0.04, 0.49)	0.04 (−0.23, 0.31)	−0.22 (−0.54, 0.1)	Clodronate										
**−0.44 (−0.57, −0.31)**	**−0.2 (−0.35, −0.05)**	−0.2 (−0.44, 0.04)	−0.39 (−0.63, −0.15)	**−0.65 (−0.95, −0.35)**	**−0.43 (−0.65, −0.21)**	Denosumab									
−0.03 (−0.11, 0.06)	**0.22 (0.1, 0.33)**	**0.22 (0, 0.43)**	0.03 (−0.19, 0.24)	−0.24 (−0.52, 0.04)	−0.01 (−0.21, 0.18)	**0.42 (0.25, 0.57)**	Etidronate								
**−0.38 (−0.56, −0.2)**	−0.14 (−0.33, 0.06)	−0.14 (−0.41, 0.13)	**−0.33 (−0.59, −0.06)**	**−0.59 (−0.91, −0.27)**	**−0.37 (−0.62, −0.12)**	0.06 (−0.16, 0.28)	**−0.35 (−0.55, −0.16)**	Ibandronate							
**−0.05 (−0.19, 0.09)**	**0.2 (0.03, 0.35)**	0.19 (−0.05, 0.43)	0 (−0.24, 0.24)	−0.26 (−0.56, 0.04)	−0.04 (−0.27, 0.19)	**0.39 (0.19, 0.59)**	−0.02 (−0.19, 0.14)	**0.33 (0.1, 0.56)**	Pamidronate						
**−0.2 (−0.4, 0)**	0.04 (−0.17, 0.26)	0.04 (−0.25, 0.32)	−0.15 (−0.44, 0.13)	**−0.41 (−0.75, −0.08)**	−0.19 (−0.46, 0.08)	0.24 (−0.01, 0.48)	−0.18 (−0.4, 0.04)	0.18 (−0.09, 0.45)	−0.15 (−0.4, 0.09)	Raloxifene					
−0.1 (−0.22, 0.02)	**0.15 (0, 0.28)**	0.15 (−0.09, 0.37)	−0.04 (−0.28, 0.18)	**−0.31 (−0.6, −0.02)**	−0.08 (−0.3, 0.13)	**0.35 (0.16, 0.52)**	−0.07 (−0.22, 0.07)	**0.28 (0.06, 0.49)**	−0.05 (−0.24, 0.14)	0.11 (−0.13, 0.34)	Risedronate				
−0.06 (−0.24, 0.12)	0.18 (−0.01, 0.38)	0.18 (−0.09, 0.45)	−0.01 (−0.28, 0.26)	−0.27 (−0.59, 0.05)	−0.05 (−0.3, 0.21)	**0.38 (0.15, 0.61)**	−0.03 (−0.24, 0.17)	**0.32 (0.06, 0.58)**	−0.01 (−0.24, 0.22)	0.14 (−0.13, 0.42)	0.04 (−0.18, 0.26)	Simvastatin			
−0.2 (−0.44, 0.04)	0.05 (−0.2, 0.29)	0.05 (−0.26, 0.35)	−0.14 (−0.45, 0.16)	**−0.41 (−0.77, −0.05)**	−0.18 (−0.48, 0.12)	0.25 (−0.03, 0.52)	−0.17 (−0.43, 0.09)	0.18 (−0.11, 0.48)	−0.15 (−0.43, 0.13)	0.01 (−0.31, 0.32)	−0.1 (−0.37, 0.17)	−0.14 (−0.44, 0.17)	Teriparatide		
**−0.21 (−0.4, −0.02)**	0.03 (−0.15, 0.21)	0.03 (−0.24, 0.29)	−0.16 (−0.42, 0.1)	**−0.42 (−0.75, −0.09)**	−0.2 (−0.46, 0.06)	0.23 (−0.01, 0.46)	−0.19 (−0.4, 0.03)	0.17 (−0.09, 0.43)	−0.16 (−0.4, 0.08)	−0.01 (−0.29, 0.27)	−0.12 (−0.34, 0.12)	−0.15 (−0.42, 0.12)	−0.02 (−0.32, 0.29)	Teriparatide + alendronate	
**−0.12 (−0.22, −0.01)**	**0.13 (0, 0.25)**	0.12 (−0.1, 0.35)	−0.07 (−0.29, 0.16)	−0.33 (−0.62, −0.04)	−0.11 (−0.31, 0.1)	**0.32 (0.15, 0.49)**	−0.09 (−0.23, 0.05)	**0.26 (0.06, 0.47)**	−0.07 (−0.25, 0.11)	0.08 (−0.14, 0.32)	−0.02 (−0.18, 0.15)	−0.06 (−0.27, 0.16)	0.08 (−0.19, 0.34)	0.09 (−0.13, 0.31)	Zoledronic acid

### BMD at 12 months

**FIGURE 6:**
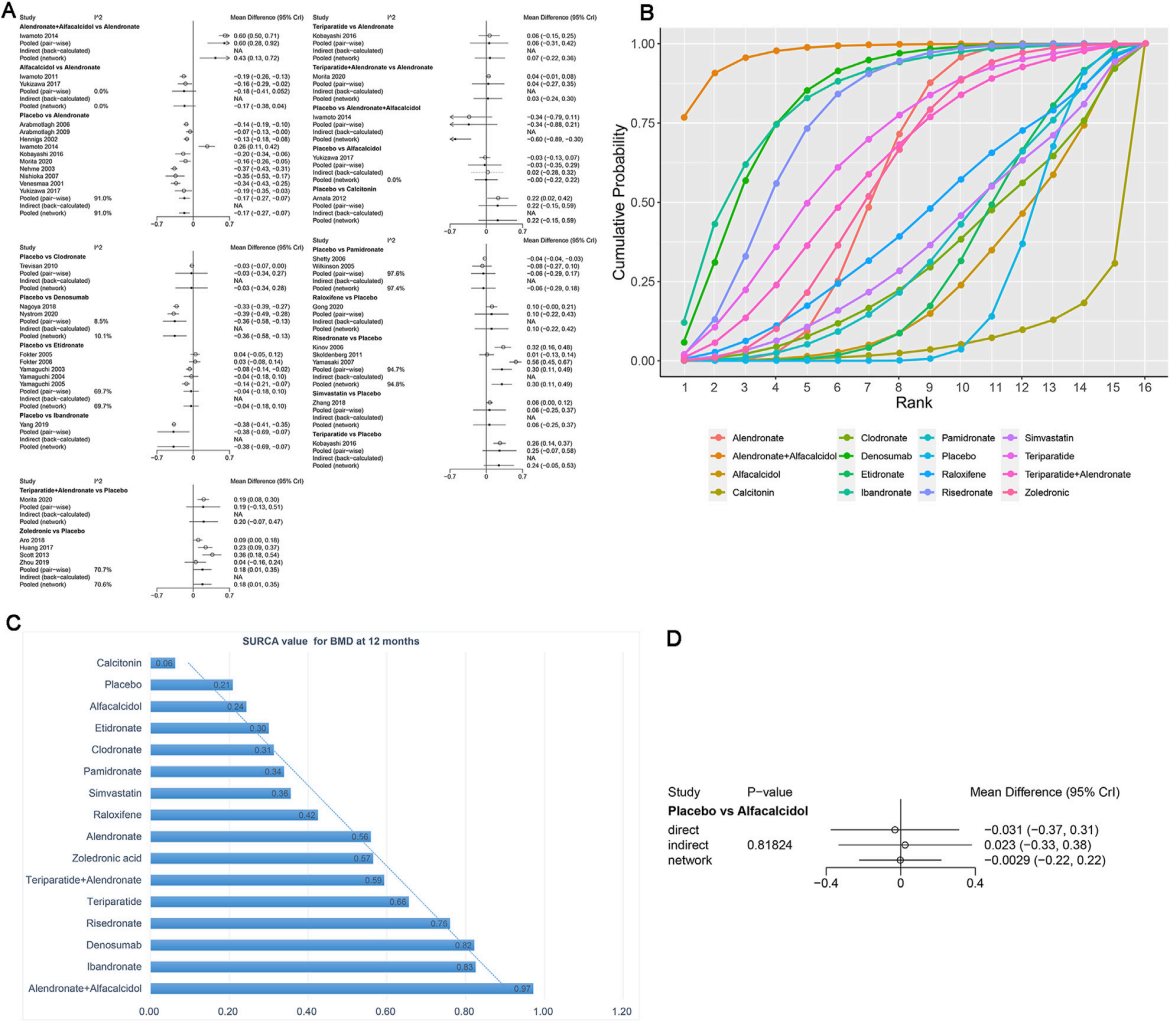
**(A)** Heterogeneity between different comparisons of drugs for BMD at 12 months. **(B)** Surface under the cumulative ranking curve (SUCRA) probabilities of different drugs for BMD at 12 months. **(C)** SUCRA values of the different drugs for BMD at 12 months. **(D)** Node-splitting method in comparisons between direct and indirect evidence for BMD at 12 months.

The network meta-analysis demonstrated that, compared with the control group, alendronate (WMD = 0.17, 95% CrI: 0.07, 0.27), alendronate + alfacalcidol (WMD = 0.6, 95% CrI: 0.29, 0.89), denosumab (WMD = 0.36, 95% CrI: 0.13, 0.58), ibandronate (WMD = 0.38, 95% CrI: 0.07, 0.69), risedronate (WMD = −0.30, 95% CrI: −0.49, -0.11), and zoledronic acid (WMD = −0.18, 95% CrI: −0.35, -0.01) exhibited a beneficial role in increasing the BMD at 12 months (Table 3). Figure 6A summarizes the heterogeneity between different comparisons of drugs. There was high heterogeneity between alendronate and placebo (I^2^ = 91.0%), etidronate and placebo (I^2^ = 69.7%), zoledronic acid and placebo (I^2^ = 70.6%), pamidronate and placebo (I^2^ = 97.4%), and risedronate and placebo (I^2^ = 94.7%). Thus, we applied the random effect model for the cumulative values.

**TABLE 3:** Efficacy of different comparisons of drugs by WMDs and corresponding 95% CrIs for BMD at 12 months; Bold fonts indicate p-value <0.05.

Placebo															
**−0.17 (−0.27, −0.07)**	Alendronate														
**−0.6 (−0.89, −0.29)**	**−0.43 (−0.71, −0.13)**	Alendronate + alfacalcidol													
0 (−0.22, 0.22)	0.17 (−0.04, 0.38)	**0.6 (0.23, 0.95)**	Alfacalcidol												
0.22 (−0.15, 0.59)	**0.39 (0.01, 0.77)**	**0.82 (0.34, 1.28)**	0.22 (−0.21, 0.65)	Calcitonin											
−0.03 (−0.34, 0.28)	0.14 (−0.19, 0.46)	**0.57 (0.13, 0.99)**	−0.03 (−0.41, 0.35)	−0.25 (−0.73, 0.23)	Clodronate										
**−0.36 (−0.58, −0.13)**	−0.19 (−0.43, 0.06)	0.24 (−0.14, 0.61)	**−0.35 (−0.67, −0.04)**	**−0.58 (−1.01, −0.15)**	−0.32 (−0.71, 0.06)	Denosumab									
−0.04 (−0.18, 0.1)	0.13 (−0.04, 0.31)	**0.56 (0.22, 0.88)**	−0.04 (−0.3, 0.23)	−0.26 (−0.65, 0.13)	−0.01 (−0.35, 0.33)	**0.32 (0.05, 0.59)**	Etidronate								
**−0.38 (−0.69, −0.07)**	−0.21 (−0.54, 0.12)	0.22 (−0.22, 0.64)	**−0.38 (−0.76, 0)**	**−0.6 (−1.08, −0.12)**	−0.35 (−0.78, 0.09)	−0.02 (−0.41, 0.36)	**−0.34 (−0.68, 0)**	Ibandronate							
−0.06 (−0.29, 0.18)	0.12 (−0.14, 0.37)	**0.54 (0.16, 0.91)**	−0.05 (−0.37, 0.27)	−0.28 (−0.71, 0.16)	−0.02 (−0.41, 0.36)	0.3 (−0.02, 0.63)	−0.02 (−0.29, 0.26)	0.32 (−0.06, 0.71)	Pamidronate						
−0.1 (−0.43, 0.22)	0.07 (−0.27, 0.41)	**0.5 (0.05, 0.93)**	−0.1 (−0.49, 0.29)	−0.32 (−0.81, 0.17)	−0.07 (−0.52, 0.38)	0.25 (−0.14, 0.65)	−0.06 (−0.42, 0.29)	0.28 (−0.17, 0.73)	−0.05 (−0.45, 0.35)	Raloxifene					
**−0.3 (−0.49, −0.11)**	−0.13 (−0.35, 0.09)	0.3 (−0.06, 0.65)	**−0.3 (−0.59, 0)**	**−0.52 (−0.93, −0.11)**	−0.27 (−0.64, 0.1)	0.06 (−0.24, 0.36)	**−0.26 (−0.5, −0.02)**	0.08 (−0.29, 0.45)	−0.25 (−0.55, 0.06)	−0.2 (−0.58, 0.18)	Risedronate				
−0.06 (−0.37, 0.25)	0.11 (−0.22, 0.44)	**0.54 (0.1, 0.96)**	−0.06 (−0.44, 0.32)	−0.28 (−0.76, 0.2)	−0.03 (−0.47, 0.41)	0.3 (−0.09, 0.69)	−0.02 (−0.37, 0.33)	0.32 (−0.12, 0.76)	0 (−0.39, 0.39)	0.04 (−0.41, 0.49)	0.24 (−0.13, 0.61)	Simvastatin			
−0.24 (−0.53, 0.05)	−0.07 (−0.36, 0.22)	0.36 (−0.06, 0.76)	−0.24 (−0.59, 0.11)	−0.46 (−0.93, 0.01)	−0.21 (−0.63, 0.21)	0.11 (−0.25, 0.48)	−0.2 (−0.53, 0.12)	0.14 (−0.29, 0.56)	−0.19 (−0.56, 0.18)	−0.14 (−0.57, 0.29)	0.06 (−0.29, 0.4)	−0.18 (−0.61, 0.24)	Teriparatide		
−0.2 (−0.48, 0.08)	−0.03 (−0.31, 0.25)	0.4 (−0.01, 0.79)	−0.2 (−0.54, 0.14)	−0.42 (−0.88, 0.04)	−0.17 (−0.58, 0.25)	0.16 (−0.2, 0.51)	−0.16 (−0.47, 0.15)	0.18 (−0.24, 0.59)	−0.14 (−0.5, 0.22)	−0.1 (−0.53, 0.33)	0.1 (−0.24, 0.44)	−0.14 (−0.56, 0.28)	0.04 (−0.35, 0.43)	Teriparatide + alendronate	
**−0.18 (−0.35, −0.01)**	−0.01 (−0.21, 0.19)	**0.42 (0.07, 0.76)**	−0.17 (−0.45, 0.1)	−0.4 (−0.8, 0.01)	−0.15 (−0.5, 0.21)	0.18 (−0.1, 0.46)	−0.14 (−0.36, 0.09)	0.2 (−0.15, 0.56)	−0.12 (−0.41, 0.17)	−0.08 (−0.44, 0.29)	0.12 (−0.14, 0.38)	−0.12 (−0.48, 0.24)	0.06 (−0.27, 0.4)	0.02 (−0.3, 0.35)	Zoledronic acid

As shown in Figures 6B,C, the SUCRA values of the five interventions demonstrated that alendronate + alfacalcidol was most effective for BMD at 12 months (SUCRA = 0.97), followed by ibandronate (SUCRA = 0.83) and denosumab (SUCRA = 0.82).

We used the node-splitting method and its Bayesian p-value to report the inconsistency of our results. The confidence intervals from direct and indirect evidence are, in general, consistent, with minor differences (Figure 6D, p = 0.82).

### BMD at 24 months

**FIGURE 7:**
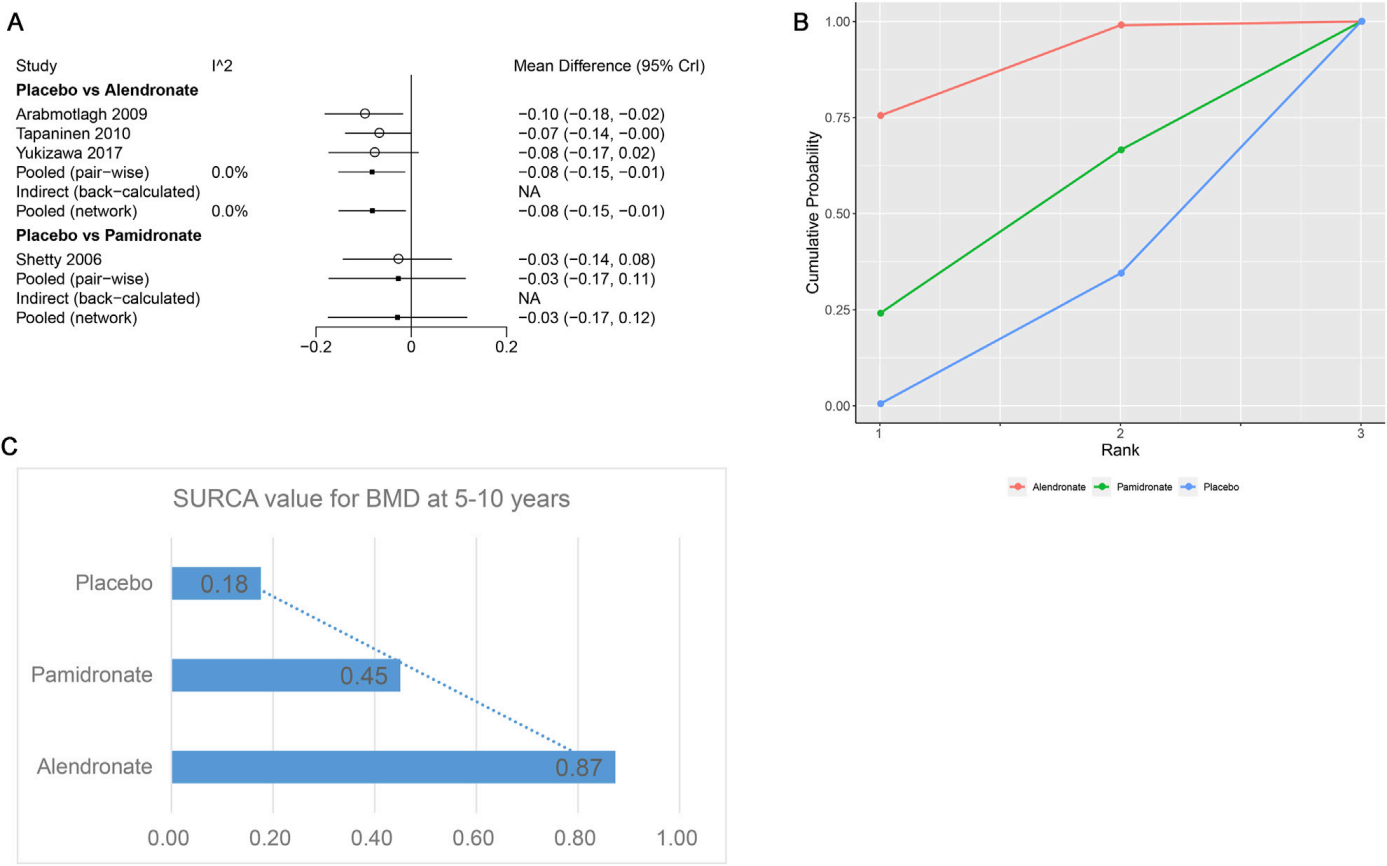
**(A)** Heterogeneity between different comparisons of drugs for BMD at 24 months. **(B)** Surface under the cumulative ranking curve (SUCRA) probabilities of different drugs for BMD at 24 months. **(C)** SUCRA values of the different drugs for BMD at 24 months.

Sixteen studies that examined nine drugs (alendronate, alfacalcidol, denosumab, etidronate, pamidronate, raloxifene, risedronate, teriparatide + alendronate, zoledronic acid, and placebo) were included in the analysis regarding BMD at 24 months. According to our results, patients using teriparatide + alendronate had the highest BMD at 24 months compared to placebo (teriparatide + alendronate: WMD = 0.17, 95% CrI 0.08, 0.26; denosumab: WMD = 0.17, 95% CrI 0.04, 0.30; alendronate: WMD = 0.12, 95% CrI 0.07, 0.18; raloxifene: WMD = 0.16, 95% CrI 0.04, 0.29, zoledronic acid: WMD = 0.14, 95% CrI 0.07, 0.21, Table 4). There was no heterogeneity between the included treatments. Thus, we applied the fixed effect model for the cumulative values (Figure 7A).

**TABLE 4:** Efficacy of different comparisons of drugs by WMDs and corresponding 95% CrIs for BMD at 24 months. Bold fonts indicate p-value <0.05.

Placebo									
**−0.12 (−0.18, −0.07)**	Alendronate								
−0.02 (−0.13, 0.1)	0.11 (−0.01, 0.22)	Alfacalcidol							
**−0.17 (−0.3, −0.04)**	−0.05 (−0.19, 0.09)	−0.16 (−0.33, 0.02)	Denosumab						
−0.06 (−0.32, 0.19)	0.06 (−0.2, 0.32)	−0.05 (−0.33, 0.24)	0.11 (−0.18, 0.39)	Etidronate					
−0.05 (−0.13, 0.04)	0.08 (−0.02, 0.18)	−0.03 (−0.17, 0.11)	0.13 (−0.03, 0.28)	0.02 (−0.25, 0.28)	Pamidronate				
**−0.16 (−0.29, −0.04)**	−0.04 (−0.18, 0.1)	−0.15 (−0.32, 0.02)	0.01 (−0.17, 0.19)	−0.1 (−0.39, 0.18)	−0.12 (−0.27, 0.04)	Raloxifene			
−0.04 (−0.13, 0.05)	0.08 (−0.02, 0.19)	−0.03 (−0.17, 0.12)	0.13 (−0.02, 0.29)	0.02 (−0.25, 0.29)	0.01 (−0.11, 0.13)	0.12 (−0.03, 0.28)	Risedronate		
**−0.17 (−0.26, −0.08)**	−0.04 (−0.13, 0.04)	**−0.15 (−0.29, −0.02)**	0 (−0.16, 0.16)	−0.1 (−0.37, 0.16)	**−0.12 (−0.24, 0)**	0 (−0.16, 0.15)	**−0.13 (−0.25, −0.01)**	Teriparatide + alendronate	
**−0.14 (−0.21, −0.07)**	−0.02 (−0.11, 0.08)	−0.12 (−0.26, 0.01)	0.03 (−0.12, 0.18)	−0.08 (−0.34, 0.19)	−0.09 (−0.21, 0.02)	0.02 (−0.12, 0.17)	**−0.1 (−0.21, 0.01)**	0.03 (−0.09, 0.14)	Zoledronic acid

As shown in Figures 7B,C, the SUCRA values of the ten interventions demonstrated that teriparatide + alendronate was most effective for BMD at 24 months (SUCRA = 0.82), followed by raloxifene (SUCRA = 0.77) and denosumab (SUCRA = 0.79).

### BMD at 5–10 years

**FIGURE 8:**
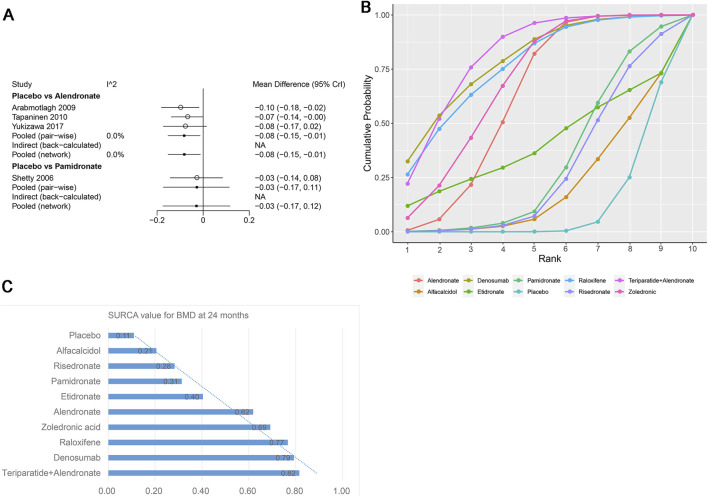
**(A)** Heterogeneity between different comparisons of drugs for BMD at 5–10 years. **(B)** Surface under the cumulative ranking curve (SUCRA) probabilities of different drugs for BMD at 5–10 years. **(C)** SUCRA values of the different drugs for BMD at 5–10 years.

Four studies examining two drugs (alendronate, pamidronate, and placebo) were involved in the analysis regarding BMD at 5–10 years. According to our results, patients using alendronate had the highest BMD at 5–10 years compared to other drugs (alendronate: WMD = 0.08, 95% CrI 0.01, 0.15; pamidronate: WMD = 0.03, 95% CrI −0.11, 0.17, Table 5). There was no heterogeneity between the included treatments (Figure 8A). Thus, we applied the fixed-effect model for the cumulative values. As shown in Figures 8B,C, the SUCRA values of the two interventions demonstrated that alendronate achieved the highest SUCRA values of the BMD at 5–10 years (0.87).

**TABLE 5:** Efficacy of different comparisons of drugs by WMDs and corresponding 95% CrIs for BMD at 5–10 years. Bold fonts indicate p-value <0.05.

Placebo		
−0.08 (−0.15, −0.01)	Alendronate	
−0.03 (−0.17, 0.11)	0.05 (−0.11, 0.21)	Pamidronate

### Publication bias and meta-regression

**FIGURE 9:**
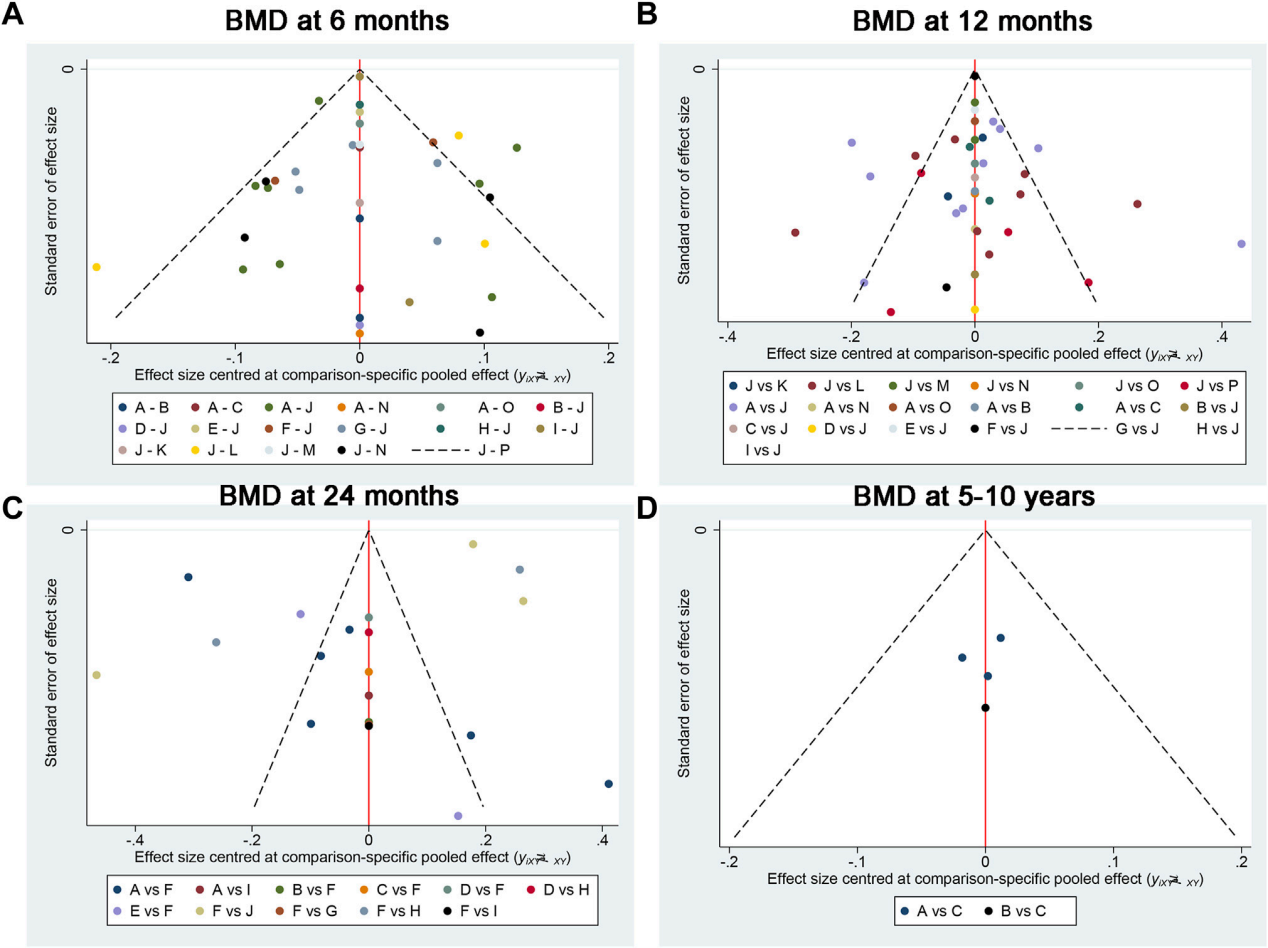
Publication bias between the included studies for **(A)** BMD at 6 months, **(B)** BMD at 12 months, **(C)** BMD at 24 months, and **(D)** BMD at 5–10 years. A, alendronate; B, alendronate + alfacalcidol; C, alfacalcidol; D, calcitonin; E, clodronate; F, denosumab; G, etidronate; H, ibandronate; I, pamidronate; J, raloxifene; K, risedronate; L, simvastatin; M, teriparatide; N, teriparatide + alendronate; O, zoledronic acid; H, placebo.

Figure 9 exhibited that there was no publication bias in the included studies. We conducted meta-regression analyses on BMD at 6 months, 12 months, 24 months, and 5–10 years according to the publication year, age, sample size, prosthesis type (cemented or uncemented), and with or without osteoporosis. We noted that the conclusions on the outcomes did not change substantially after accounting for potential effect modifiers (Table 6).

**TABLE 6:** Meta-regression analysis of the outcomes.

	Covariates	b	95% credible interval of b
BMD at 6 months	Publication year	−0.1175689	−0.27098; 0.03851
	Age	−0.000206	−0.095823; 0.09480
	Sample size	0.011683	−0.124224; 0.1468740
	Cemented or uncemented	−0.004299	−0.097810; 0.091263
	Osteoporosis or not	0.008533	−0.063521; 0.146322
BMD at 12 months	Publication year	−0.15858	−0.333930; 0.009685
	Age	−0.0009352	−0.1541726; 0.1628
	Sample size	−0.139331	−0.45853; 0.1613
	Cemented or uncemented	−0.02821	−0.207217; 0.1179
	Osteoporosis or not	−0.15332	−0.35261; 0.18612
BMD at 24 months	Publication year	0.062848	−0.0378921; 0.16038
	Age	0.01632	−0.078535; 0.1108
	Sample size	0.05965	−0.184667; 0.33025
	Cemented or uncemented	0.01599	−0.097171; 0.12792
	Osteoporosis or not	0.03621	−0.00632; 0.53278
BMD at 5–10 years	Publication year	−0.005476	−0.146044; 0.13345
	Age	−0.01891	−0.155774; 0.11487
	Sample size	−0.01578	−0.163547; 0.12876
	Cemented or uncemented	−0.07346	−2.220064; 0.81534
	Osteoporosis or not	−0.00362	−3.134332,0.753217

## Discussion

### Main findings

This is the first systematic review with network meta-analysis (NMA) comparing the effects of different anti-osteoporosis drugs on femoral periprosthetic bone loss following THA. By synthesizing data with the highest level of evidence, we found that both denosumab and bisphosphonates can effectively prevent bone loss after THA. Denosumab was identified as the most effective agent for increasing BMD at 6 months post-THA. Alendronate, when combined with alfacalcidol or teriparatide, was found to be the most effective for increasing BMD at 12 months and 24 months after THA.

### Compared with previous meta-analyses

The present study is the first systematic review and network meta-analysis that compared anti-osteoporotic drugs on the preservation of periprosthetic BMD followed-up for long-term efficiency after THA. Our findings both align with and extend the conclusions of earlier traditional meta-analyses. Previous meta-analyses, such as those by Shi et al. (2018b) and Zhao et al. (2015), consistently demonstrated that bisphosphonates are effective in reducing periprosthetic bone loss after THA compared to placebo. Our results confirm the efficacy of bisphosphonates, particularly alendronate and zoledronic acid, across various time points.

However, a key advancement of our network meta-analysis is the ability to rank multiple treatments simultaneously, including newer agents like denosumab and combination therapies. While prior meta-analyses primarily focused on bisphosphonate-versus-placebo comparisons, our NMA reveals that denosumab may offer superior early BMD preservation compared to individual bisphosphonates. This finding does not necessarily contradict previous studies but rather refines and expands upon them by incorporating a broader spectrum of interventions. For instance, the superior ranking of combination therapies (alendronate + alfacalcidol at 12 months and teriparatide + alendronate at 24 months) highlights potential synergistic effects that were not extensively evaluated in earlier pairwise meta-analyses due to the scarcity of head-to-head trials.

Our analysis regarding certain drugs like clodronate, etidronate, pamidronate, and risedronate, which showed no significant difference from placebo in some comparisons, underscores the heterogeneity in efficacy among different bisphosphonates, a nuance that could not be fully elucidated in traditional meta-analyses that often grouped them together. Therefore, rather than contradicting previous findings, this NMA provides a more granular and hierarchical perspective on the comparative effectiveness of anti-osteoporosis medications for this specific clinical indication, leveraging both direct and indirect evidence to inform treatment choices more precisely.

Our primary finding is that denosumab most significantly preserves periprosthetic BMD among anti-osteoporotic drugs (SUCRA values at 6 months, 12 months, and more than 2 years postoperatively were 97.1%, 82.2%, and 79.2%, respectively). Several large clinical trials have shown the advantages of denosumab compared with bisphosphonates in postmenopausal women (Suzuki et al., 2018a; Suzuki et al., 2018b; Nakamura et al., 2017). Treatment with denosumab illustrated that the early migration of the tibial component had reduced in total knee arthroplasty, as determined using radiostereometric analysis (Ledin et al., 2017). Nystrom et al. (Ledin et al., 2017) reported that denosumab potently prevents early periprosthetic bone loss after uncemented THA by measuring periprosthetic BMD by DXA. They analyzed the periprosthetic standardized uptake value (SUV) by [^18^F] sodium fluoride (18F NaF) positron emission tomography/CT (F-PET). Our study found that denosumab showed a significant increase in the preservation efficiency compared with bisphosphonates, such as alendronate, zoledronate, pamidronate, etidronate, and risedronate. The pronounced efficacy of denosumab in preserving periprosthetic bone mineral density (BMD) at 6 months post-THA can be attributed to its distinct mechanism of action.

Denosumab is a fully human monoclonal antibody that specifically targets the receptor activator of nuclear factor kappa B ligand (RANKL) (Aapro et al., 2025). By binding to RANKL, denosumab prevents its interaction with the RANK receptor on osteoclast precursors and mature osteoclasts, thereby inhibiting osteoclast formation, function, and survival (Singh et al., 2025). This rapid and direct blockade of the RANK/RANKL pathway results in a swift reduction in bone resorption. Unlike bisphosphonates, which must be incorporated into the bone matrix and internalized by osteoclasts to induce apoptosis, denosumab acts systemically and does not require bone binding, allowing for a more immediate antiresorptive effect. This rapid onset of action likely explains its superior performance in the early postoperative period, a critical phase characterized by accelerated periprosthetic bone turnover due to surgical trauma and adaptive bone remodeling.

Alendronate had the third-highest ranking at 12 months and 24 months after THA. Several studies reported that alendronate reduced periprosthetic bone loss after THA (Nishioka et al., 2007; Yukizawa et al., 2017; Venesmaa et al., 2001). Zoledronate was more effective than placebo at all follow-up time points. The loss of periprosthetic BMD after THA could be effectively reversed using zoledronate (Zhou et al., 2019). Clodronate, etidronate, pamidronate, and risedronate showed no significant difference compared with placebo in our network analysis. Iwamoto et al. confirmed that alfacalcidol did not show any effects in any regions after hybrid-type THA (Iwamoto et al., 2011).

Teriparatide is another promising potent drug in terms of preventing periprosthetic bone loss after THA (SUCRA values at 6 months and 12 months postoperatively were 63.3% and 65.5%, respectively). Kobayashi et al. (2016) reported that the application of teriparatide alone showed good protection results after THA for osteoporotic patients. Switching administration of teriparatide before alendronate had a significant effect on BMD of the lumbar spine and zones 1 and 7 at 2 years postoperatively, and the study found that the combination was more effective than alendronate alone (Morita et al., 2020). However, in our network analysis, there was no significant difference between teriparatide and alendronate. More high-quality trials comparing two drugs are needed for a solid result.

Patients taking raloxifene reported higher improvement in periprosthetic BMD for postmenopausal women compared with placebo at 24 months postoperatively (Gong et al., 2020). SUCRA values at 6 months, 12 months, and more than 2 years postoperatively were 83.1%, 58.5% and 83.9%, respectively. Therefore, raloxifene is possibly well suited to prevent periprosthetic bone loss after THA in postmenopausal women.

Calcitonin was even worse than a placebo, and the reduction was significantly different at 12 months postoperatively. Arnala (2012) reported that Nasal salmon calcitonin 200 IU on a daily basis did not promote any additional calcium substitution to prevent bone loss after hip replacement. It seems that calcitonin does not promote any BMD added value to prevent bone loss after THA.

While this network meta-analysis demonstrates the efficacy of several pharmacologic interventions in preserving periprosthetic BMD, the ultimate clinical goal is to prevent adverse events such as aseptic loosening and periprosthetic fractures. Our review of the available data on these hard endpoints highlights a critical gap in the current literature. The included RCTs were primarily designed and powered to detect differences in BMD and lacked the long-term follow-up necessary to assess rare clinical events. Consequently, while denosumab and bisphosphonates show significant benefits for BMD preservation, the current evidence is insufficient to confirm that these benefits directly translate to a reduced risk of loosening or fracture. The pathway from reduced bone loss to improved implant survival is protracted and multifactorial. Future research prioritizing long-term follow-up and the analysis of large-scale registry data is essential to validate the effect of anti-osteoporosis treatments on these critical patient-centered outcomes.

There are several limitations related to the inferences provided in the present network meta-analysis. First, the baseline characteristics of the included trials are various, including gender, ages of patients, primary diseases, type of prosthesis, and dose of medicine, which would lead to bias. Second, the analysis of long-term efficacy (5–10 years) is based on a very sparse network, including only two active drugs (alendronate and pamidronate) from four studies. The ranking of alendronate as the best intervention for this time point should therefore not be overinterpreted as it primarily reflects the scarcity of long-term comparative data rather than established clinical superiority. Third, most of the included studies involve active agents versus a placebo. The lack of head-to-head trials with large samples increases the risk of bias.

### Strengths

Despite these limitations, our study has several notable strengths. It is the first NMA to provide a comprehensive ranking of a wide array of anti-osteoporosis medications for this specific clinical application. We employed robust statistical methods to synthesize both direct and indirect evidence, thus strengthening the comparative conclusions. The analyses included multiple follow-up time points, offering valuable insights into the temporal efficacy of different treatments, which is crucial for informing clinical decision-making regarding treatment initiation and duration.

## Conclusion

This network meta-analysis provides comparative effectiveness estimates for various anti-osteoporosis treatments aimed at preserving femoral periprosthetic BMD following THA. Our results suggest that denosumab is the most effective agent for increasing BMD at 6 months postoperatively. For the 12- and 24-month periods, combination therapies, particularly alendronate with alfacalcidol or teriparatide, appear to yield superior BMD preservation compared to monotherapies. However, it is important to note that these findings regarding combination regimens are based on a limited number of studies and thus should be interpreted with caution until further high-quality direct-comparison trials are available. Based on the current evidence, a potential clinical strategy could involve initiating denosumab within the first 6 months after THA to capitalize on its early efficacy, followed by transition to a combination regimen such as alendronate with alfacalcidol or teriparatide for mid- to long-term management, especially in patients at high risk of periprosthetic bone loss. Nonetheless, the optimal timing, sequence, and patient selection for such therapeutic approaches remain to be established. Future research should prioritize head-to-head RCTs comparing active treatments, standardize outcome reporting, and include longer follow-up durations to better inform clinical decision-making and treatment guidelines.

## Data Availability

The original contributions presented in the study are included in the article/Supplementary Material, further inquiries can be directed to the corresponding author.

## Supplementary material

SUPPLEMENTARY FIGURE 1 Demographic characteristics for all treatments. **(A)** Number of randomized for BMD at 6 months; **(B)** Publication year for BMD at 6 months; **(C)** Mean age for BMD at 6 months; **(D)** Number of randomized for BMD at 12 months; **(E)** Publication year for BMD at 12 months; **(F)** Mean age for BMD at 12 months; **(G)** Number of randomized for BMD at 24 months; **(H)** Publication year for BMD at 24 months; **(I)** Mean age for BMD at 24 months; **(J)** Number of randomized for BMD at 5–10 years; **(K)** Publication year for BMD at 5–10 years; **(L)** Mean age for BMD at 5–10 years.
