# Supplementary figures and images for: Effectiveness of treatments to prevent femoral periprosthetic bone loss following total hip arthroplasty: a network meta-analysis
Source: Front Pharmacol. 2025 Nov 27;16:1566890. doi: 10.3389/fphar.2025.1566890 (PMC12695822; doi:10.3389/fphar.2025.1566890)

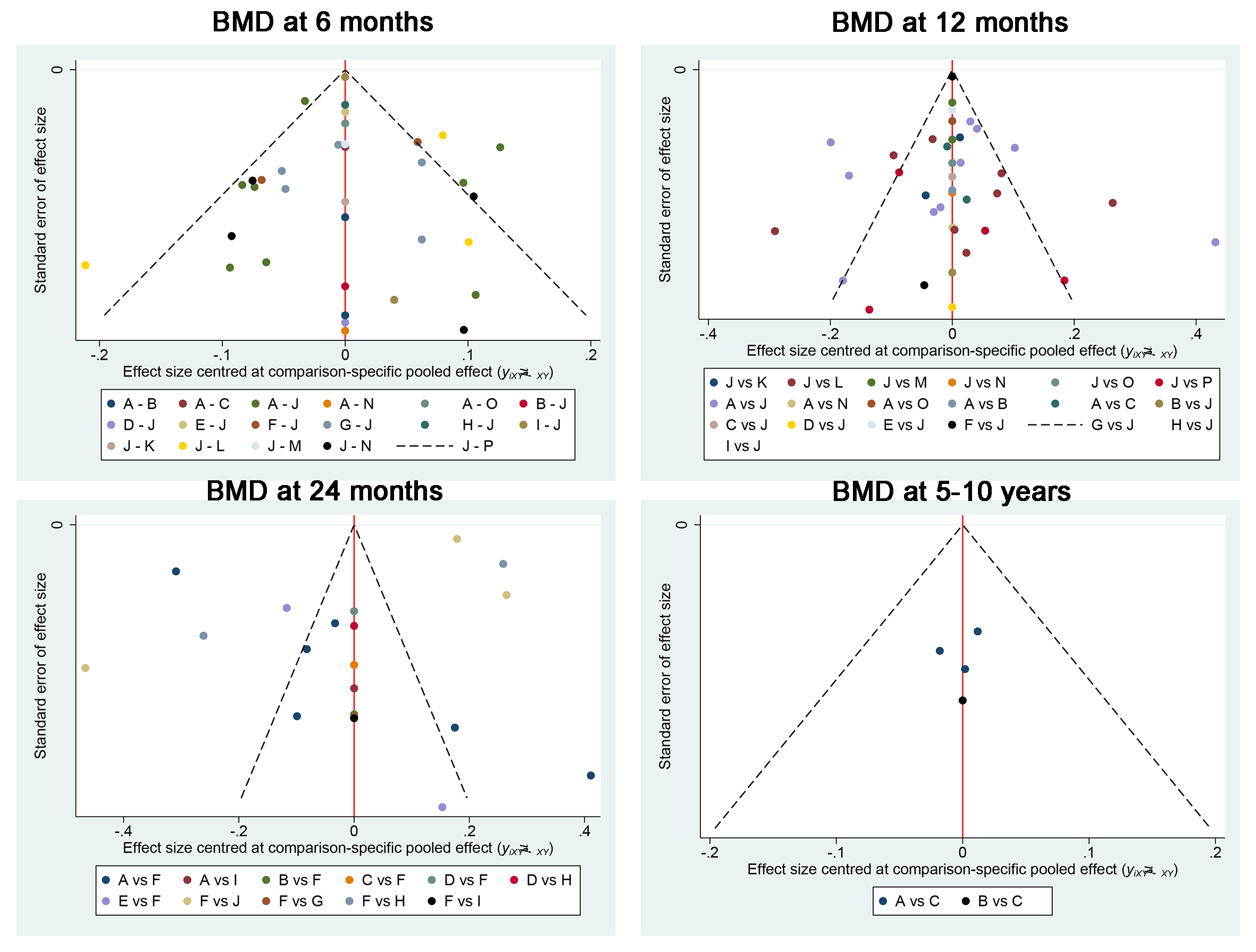

Supplement: Supplementary file 1 [file Image2.tif]

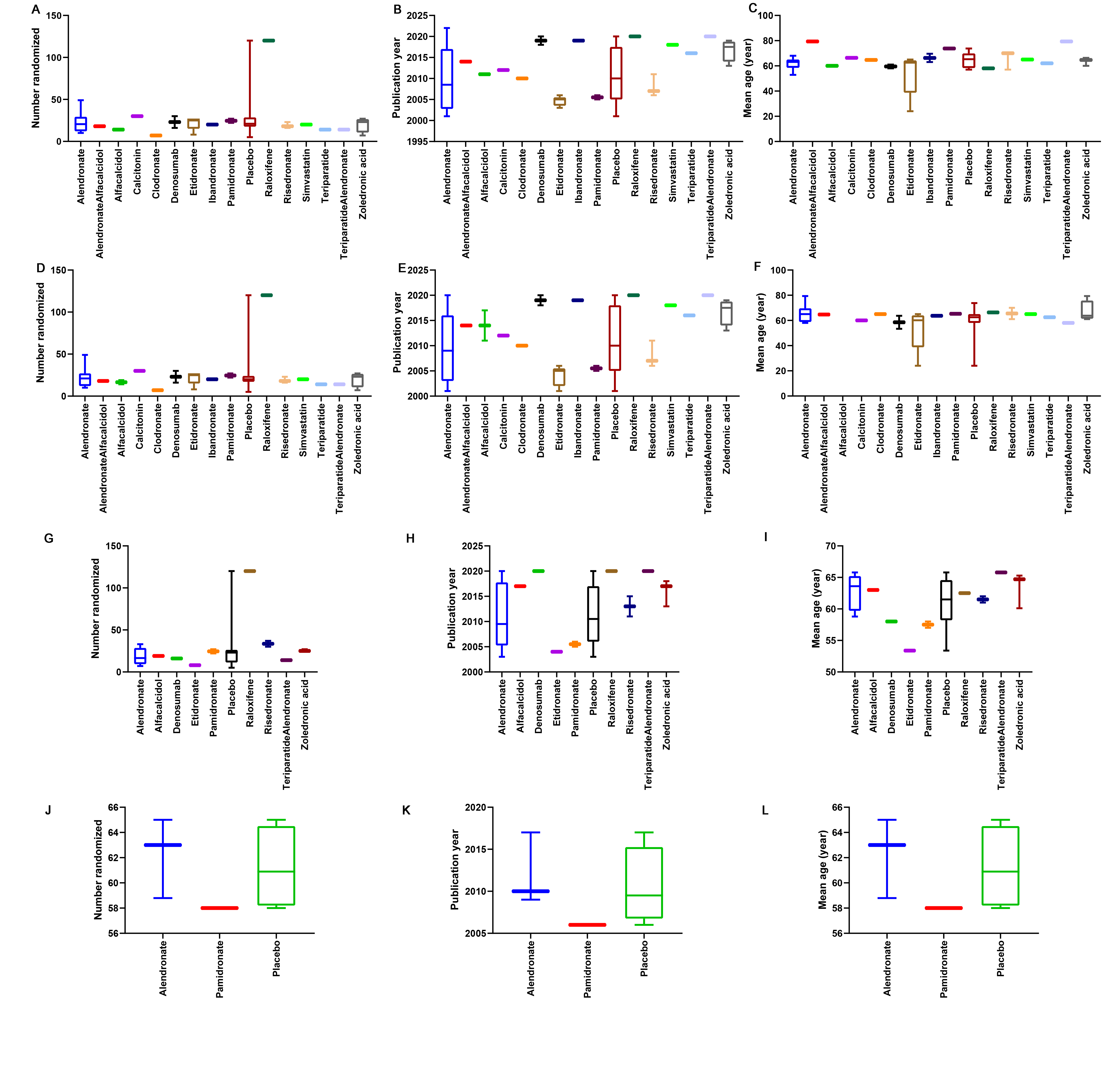

Supplement: Supplementary file 2 [file Image1.tif]
